# Prediction of Decline in Global Cognitive Function Using Machine Learning with Feature Ranking of Gait and Physical Fitness Outcomes in Older Adults

**DOI:** 10.3390/ijerph182111347

**Published:** 2021-10-28

**Authors:** Byungjoo Noh, Hyemin Yoon, Changhong Youm, Sangjin Kim, Myeounggon Lee, Hwayoung Park, Bohyun Kim, Hyejin Choi, Yoonjae Noh

**Affiliations:** 1 Department of Kinesiology, Jeju National University, Jeju 63243, Korea; bnoh@jejunu.ac.kr; 2 Department of Management Information Systems, Dong-A University, Busan 49315, Korea; yoonlea1205@donga.ac.kr (H.Y.); kar98n@donga.ac.kr (Y.N.); 3 Department of Health Sciences, The Graduate School of Dong-A University, Busan 49315, Korea; app00113@donga.ac.kr (H.P.); 2177638@donga.ac.kr (B.K.); chjin0907@donga.ac.kr (H.C.); 4 Department of Health and Human Performance, University of Houston, Houston, TX 77004, USA; mlee47@central.uh.edu

**Keywords:** aging, gait analysis, physical fitness, dementia, machine learning, inertial measurement unit, global cognitive function

## Abstract

Gait and physical fitness are related to cognitive function. A decrease in motor function and physical fitness can serve as an indicator of declining global cognitive function in older adults. This study aims to use machine learning (ML) to identify important features of gait and physical fitness to predict a decline in global cognitive function in older adults. A total of three hundred and six participants aged seventy-five years or older were included in the study, and their gait performance at various speeds and physical fitness were evaluated. Eight ML models were applied to data ranked by the *p*-value (LP) of linear regression and the importance gain (XI) of XGboost. Five optimal features were selected using elastic net on the LP data for men, and twenty optimal features were selected using support vector machine on the XI data for women. Thus, the important features for predicting a potential decline in global cognitive function in older adults were successfully identified herein. The proposed ML approach could inspire future studies on the early detection and prevention of cognitive function decline in older adults.

## 1. Introduction

Gait is the end product of the neuromusculoskeletal system, which executes a desired movement by using sensory inputs to modify motor patterns and muscular output [1]. The gait pattern is commonly generated by central pattern generators (CPGs), which are the source of tightly-coupled patterns of neural activity that drive rhythmic and stereotypical motor behavior such as locomotion [2]. However, the initiation and termination of movement, direction and speed change during walking, and obstacle avoidance require a supraspinal input to make movements that are adapted to the environment by modifying the basic gait pattern [2].

Our previous studies clearly demonstrated the association between global cognitive function and motor function and gait abnormalities [3,4]. Decreased global cognitive function may be linked with increased variability and phase domains of gait in older women [3]. Furthermore, a longer stance phase of gait and physical fitness variables, such as grip strength and sit-to-stand movement, can also indicate declining global cognitive function due to aging [4]. Decreased motor function, especially gait ability, is connected to a subsequent decline in cognitive function, which can induce mild cognitive impairment and dementia [5,6]. Although several studies have investigated the association between cognitive functions and motor functions, most have only focused on walking speed, with relatively few steps [3]. In recent years, the emergence of advanced artificial intelligence and wearable sensing technology has enabled precise gait analysis, leading to more accurate analyses using machine learning (ML) techniques [7,8]. Therefore, research on gait analysis using ML algorithms can be used to determine the association between cognitive function and motor function in older adults.

For training a ML model, the higher the number of features available, the higher the number of hints that can predict the dependent variable. Although ML requires large-scale data initially, once a certain amount of research has been carried out, useful features can be selected to increase the quality of suitable data [9]. In addition, by extracting meaningful features and reducing the size of the data, learning accuracy can be improved and the results can be simplified to enable efficient analyses [10].

Therefore, the aim of this study is to identify the important features of gait and physical fitness to predict a decline in global cognitive function in older adults. The proposed ML approach can successfully identify features that indicate a potential decline in global cognitive function, and reinforced the findings obtained in previous studies.

## 2. Materials and Methods

### 2.1. Participants

The study participants were finalized based on a survey conducted in Busan, South Korea, in 2018–2019. In total, 306 adults aged 75 years or older (111 men and 195 women) were recruited through advertisements placed in the local community for the study based on the following exclusion criteria: (a) the participant is unable to walk without any support, and (b) the participant has a history of severe musculoskeletal injuries or neurophysiological issues during the previous six months. All participants provided informed consent after reading a detailed explanation of the study. The study was approved by the Institutional Review Board of Dong-A University (IRB: 2-1040709-AB-N-01-201808-HR-023-02).

### 2.2. Instrumentation

An inertial measurement unit-based (IMU-3000^TM^, InvenSense, San Jose, CA, USA) gait performance system (DynaStab^TM^, JEIOS, Busan, Korea) with shoe-type data loggers (Smart Balance SB-1^®^, JEIOS, Busan, Korea), which can be used for different-sized feet, were used herein. Gait performance data was sampled considering tri-axial acceleration (up to ±6× *g*) and angular velocities (up to ±500° s^−1^) along three orthogonal axes [11,12]. The data were recorded at a sampling frequency of 100 Hz using a data acquisition system (Smart Balance version 1.5, JEIOS, Busan, Korea).

### 2.3. Test Procedures

The biometrics (InBody 270, Biospace, Seoul, Korea) of the participants were measured before the tests. They were also asked to answer a questionnaire to assess their habitual physical activity (PA) levels; the international PA questionnaire-short form (IPAQ-SF) was used herein [13]. Prior to the tests, the participants were made to undergo a warm-up protocol, which included familiarization with the test procedure and instruments for 10 min through verbal and visual demonstrations.

#### 2.3.1. Assessment of Global Cognitive Function

To assess the global cognitive function of the participants, the Mini-Mental State Examination (MMSE) [14] was used herein. The MMSE is a 30-point questionnaire that includes orientation, attention, memory, language, and visual–spatial domains, and is the most commonly used screening tool for diagnosing dementia and assessing global cognitive function. The participants were categorized as having normal cognitive function if they had an MMSE score of 24 or more.

#### 2.3.2. Gait Performance Test at Different Speeds

The participants performed three trials of an over-ground gait performance test along a straight 20 m walkway at three different walking speeds (80% of usual walking speed (slower), 100% of usual walking speed (preferred), and 120% of usual walking speed (faster)) (see Supplementary Material Table S1) [15]. Prior to gait performance test, the participants were asked to walk on a treadmill to the estimated preferred walking speed using a metronome (beat/min), the participants continued to walk on the overground walkway as closely as possible to the three target walking speeds paced by a metronome.

#### 2.3.3. Physical Fitness Tests

Nine physical fitness tests were performed to assess four domains of physical fitness, namely, strength, flexibility, balance, and functional endurance [4,16]. The mean values of two attempts were calculated for each physical fitness test. The participants performed the following physical fitness tests (see Supplementary Material Table S1):

1. Handgrip strength (upper body strength) using a digital handgrip dynamometer (TKK 5401 Grip-D, Takei Scientific Instruments, Tokyo, Japan);
2. Dumbbell curls (upper body strength) using a dumbbell (3 kg for men and 2 kg for women);
3. Five sit-to-stand movements (lower body strength);
4. Standing time (ST) from a long sitting position (LSP) (lower body strength);
5. Back scratch (upper body flexibility);
6. Chair sit and reach (lower body flexibility);
7. Single-leg balance (dominant leg) (static balance);
8. A 3 m timed-up-and-go (TUG) (dynamic balance);
9. A 6 min walk test (functional endurance).

### 2.4. Data Analysis

The gait performance data were filtered through a second-order Butterworth low-pass filter, with a cut-off frequency of 10 Hz [11,12]. The two initial acceleration steps and two final deceleration steps were excluded to analyze the steady-state condition. The gait events (heel strikes and toe-offs) were identified based on the maximum values of the linear acceleration along the anteroposterior axis and the vertical axis during a gait cycle [11,12]. The spatiotemporal parameters, such as the walking speed, cadence, step/stride length, step/stride time, single support phase, double support phase, and stance phase, were measured, and their coefficient of variance (CV, (standard deviation/mean) × 100) values were calculated. The Z-score of all the variables (all questionnaire scores, MMSE scores, gait variables, and physical fitness variables) were calculated for normalization.

### 2.5. Feature Ranking

Feature ranking (FR) improves accuracy by reducing the inclusion of noise in the analysis by ranking the key variables that affect the dependent variable. Herein, the features were ranked by performing a marginal test with the *p*-value of simple linear regression (LP) and the XGBoost feature importance gain (XI).

#### 2.5.1. Simple Linear Regression

Simple linear regression is a linear regression model and linear function that describes the relationship between one independent variable and one dependent variable. The formula for simple linear regression is:

(1) $$y_{i}=\beta_{0}+\beta_{1}x_{i}+\epsilon$$

where, $y_{i}$ is the *i*-th response variable and $x_{i}$ is *i*-th independent variable, $\beta_{1}$ is the coefficient of the regression, $\beta_{0}$ is the intercept of the regression, and ϵ is the error of the estimate. The regression line is estimated as follows:

(2) $$\hat{y}=\hat{\beta_{0}}+\hat{\beta_{1}}\;x$$

where, $\hat{\beta_{1}}=\frac{\sum_{i=1}^{n}y_{i}x_{i}-\frac{\left(\sum_{i=1}^{n}y_{i}\right)\left(\sum_{i=1}^{n}x_{i}\right)}{n}}{\sum_{i=1}^{n}{\left(x_{i}-\bar{x}\right)}^{2}}$ and $\hat{\beta_{0}}=\bar{y}-\hat{\beta_{1}}\bar{x}$ ($\bar{y}=\frac{\sum_{i=1}^{n}y_{i}}{n}$, $\bar{x}=\frac{\sum_{i=1}^{n}x_{i}}{n}$).

The *p*-value corresponding to this statistic based on the t-distribution is [17]:

(3) $$T_{0}=\frac{{\hat{\beta }}_{1}}{se\left({\hat{\beta }}_{1}\right)}\;\mathrm{where},\;se\left(\hat{\beta_{1}}\right)=\sqrt{\frac{\sum_{i=1}^{n}{\varepsilon_{i}}^{2}}{\sum_{i=1}^{n}{\left(x_{i}-\bar{x}\right)}^{2}}}\;\mathrm{and}\;\varepsilon_{i}=y_{i}-\hat{y_{i}}$$

(4) $$p\text{-value}=2\times \left(1-P\left(T\leq T_{o}\right)\right)$$

Herein, the false discovery rate (FDR) is defined using a new *p*-value that fixes the non-significant probability among those judged to be significant. In general, FDR is defined as:

FDR = false positive/total positive (total positive = false positive + true positive)

That is, it is the ratio of data incorrectly judged to be significant to the total number of data judged to be significant. The Benjamini–Hochberg (BH) procedure was used to estimate the FDR.

(5) $$n=max\;\left\{k|\;p_{k}\leq \frac{k}{m}\alpha ,k=1,2,\ldots ,n\right\}$$

In this method, the *p*-values of all the features are sorted in descending order, and the *p*-values of each test are applied differently. The rejection range is adjusted such that it is not too conservative by fixing the FDR to 0.05, and applying increasingly larger *p*-values to features with lower *p*-values [18].

#### 2.5.2. XGBoost

XGBoost is an ensemble algorithm that uses multiple decision trees, that is, a gradient boosting decision tree [19]. The gain refers to the average gain, which is obtained by dividing the total gain of the tree by the number of divisions of each feature. Herein, the gain is used to rank the features in ascending order.

(6) $$gain=\frac{1}{2}\;\left\{\frac{{\left(\sum_{i\in R}f_{i}\right)}^{2}}{\sum_{i\in R}g_{i}+\omega }+\frac{{\left(\sum_{i\in L}f_{i}\right)}^{2}}{\sum_{i\in L}g_{i}+\omega }-\frac{{\left(\sum_{i\in I}f_{i}\right)}^{2}}{\sum_{i\in I}g_{i}+\omega }\;\right\}-\;\gamma$$

where *L* and *R* are samples of the left and right nodes, respectively, after division; I=L ∪ R; and ω and γ are the penalties for each division [20].

### 2.6. Machine Learning

#### 2.6.1. Support Vector Machine

A support vector machine (SVM) consists of hyperplanes or sets of hyperplanes that can be used for classification or regression analysis. The optimal decision boundary maximizes the margin [21].

(7) $$margin=\frac{2}{\left||a|\right|}$$

$$\arg\underset{\left(a,b\right)}{\min}\left||a|\right|,\;where\;y_{i}\;\left(\;a\;\cdot \;x_{i}-b\;\right)\;\geq 1\;\left(1\;\leq i\;\leq n\right)$$

where $x_{i}$ is an n-dimensional real vector and $y_{i}$ is a value from −1 to 1 [22].

#### 2.6.2. Decision Tree

A decision tree (DT) is an algorithm that creates predictable rules using one explanatory variable at a time as a tree structure of decision rules. The prediction proceeds such that the purity of each region increases or the entropy decreases [23].

(8) $$Entropy\left(S\right)=-\overset{n}{\underset{i=1}{\sum }}p_{i}\;\log_{2}\left(p_{i}\right)$$

where $p_{i}$ is the ratio of the *i*-th region to the variable in the *S* region.

#### 2.6.3. Random Forest

A random forest (RF) is an ensemble technique that increases the learning performance of ML by applying multiple DTs simultaneously [24]. The part created during learning repeats the same procedure until the minimum node size, which is the stopping criterion, is satisfied. The final prediction is obtained based on the average value of each tree prediction.

#### 2.6.4. Neural Network

A neural network (NN) is an artificial neural network similar to the structure of a neural network in the human brain, consisting of several hidden layers between the input layer and the output layer. Each node acts independently and has an associated transfer function that describes its weight [25].

#### 2.6.5. LASSO

Shrinkage methods reduce variance and model complexity by minimizing the residual sum of squares. Variable selection is performed by reducing the estimated regression coefficient of the predictor variable to zero, which is the process of ridge regression analysis and the least absolute shrinkage and selection operator (LASSO). Ridge regression analysis uses an L2 penalty to provide a reliable estimate when the data exhibit multicollinearity [26], whereas LASSO uses the L1 penalty function. The estimate of LASSO is as follows [27].

(9) $$\hat{\beta }=\underset{\beta }{argmin}\left\{\frac{1}{2}\overset{n}{\underset{i=1}{\sum }}{\left(y_{i}-\beta_{0}-\overset{k}{\underset{j=1}{\sum }}x_{ij}\beta_{1}\right)}^{2}+\theta_{1}\;\overset{k}{\underset{j=1}{\sum }}\left|\beta_{j}\right|\right\}$$

where $\theta_{1}$ is a parameter that controls the amount of shrinkage, and the estimated regression coefficient is truncated from 0 by transforming it according to $\theta_{1}$.

#### 2.6.6. Elastic Net

Elastic net (EN) is a linear combination of the L1 and L2 penalties. Like L1, it provides feature selection, but is not limited to sample size.

(10) $$penalty_{\theta }\left(\beta \right)=\theta_{1}{\left|\beta \right|}_{1}+\theta_{2}{\left|\beta \right|}_{2}^{2},\;\theta_{1},\;\theta_{2}\;\geq \;0$$

where $\theta_{1}$ and $\theta_{2}$ are parameters corresponding to each other. Increasing the L1 penalty reduces the number of features, whereas the L2 penalty has a grouping effect [28].

#### 2.6.7. SCAD

Smoothly clipped absolute deviation (SCAD) is a method used to estimate the regression coefficient by minimizing the least-squares function of the penalty. The SCAD penalty is implemented in the same way as the L1 penalty in the case of a small regression coefficient, whereas a large regression coefficient reduces the estimation bias by using a certain penalty instead of the L1 penalty [29,30].

(11) $$penalty_{\theta }\left(\beta \right)=\{\begin{array}{cc} \theta \left|\beta \right| & \left(\left|\beta \right|\leq \theta \right)\\ \frac{2\gamma \theta \left|\beta \right|-\beta^{2}-\theta^{2}}{2\left(\gamma -1\right)} & \left(\theta <\left|\beta \right|\leq \gamma \theta \;and\;\gamma >2\right)\\ \frac{\theta^{2}\left(\gamma +1\right)}{2} & \left(\left|\beta \right|\geq \gamma \theta \right) \end{array}$$

#### 2.6.8. MCP

The minimax concave penalty (MCP) applies the same penalty ratio as LASSO, but in the case of |β|≥γθ, it continues to relax until the penalty ratio becomes 0. MCP provides sparse convexity over a wide range by minimizing the maximum concavity [31].

(12) $$penalty_{\theta }\left(\beta \right)=\{\begin{array}{ll} \theta \left|\beta \right|-\frac{\beta^{2}}{2\gamma } & \left(\left|\beta \right|\leq \gamma \theta \right)\\ \frac{1}{2}\;\gamma \theta^{2} & \left(\left|\beta \right|\geq \gamma \theta \right) \end{array},\;\;\;\gamma >1$$

## 3. Results

**Figure 1:**
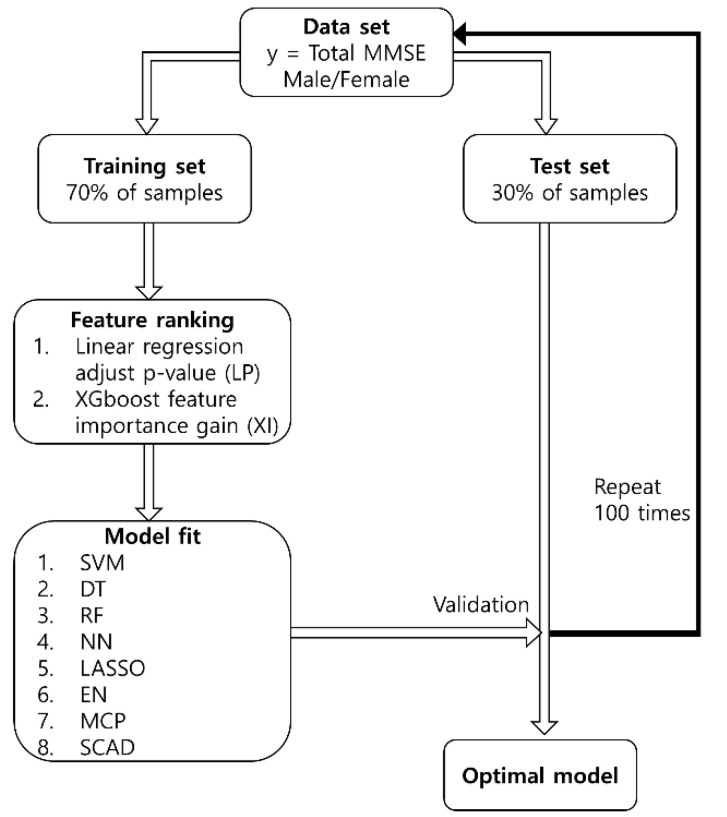
Schematic diagram of the proposed procedure. The abbreviations in the figure are: mini-mental state examination (MMSE); support vector machine (SVM); decision tree (DT); random forest (RF); neural network (NN); least absolute shrinkage and selection operator (LASSO); elastic net (EN); minimax concave penalty (MCP); smoothly clipped absolute deviation (SCAD).

To compare and analyze the variables that affect the cognitive function scores of men and women, the data were divided based on the sex of the participants. The training set comprised 70% of the samples, and the test set comprised 30% of the samples. The data comprised 74 independent variables including the sex of the participant, body information, gait variability, and spatiotemporal gait. If the data were randomly assigned, the performance may deteriorate as the variables that do not have any effect would be included as well. For each feature and cognitive function score, the LP and XI ranking in the order of 2, 3, …, 73 variables were accumulated to extract the optimal model combination with a minimum error. The procedure used herein is illustrated in Figure 1.

To ensure that the results were not biased, sampling was performed 100 times, and the root mean squared error (RMSE) was used as an error index.

(13) $$RMSE=\sqrt{\frac{\sum_{i=1}^{N}{\left(y_{i}-{\hat{y}}_{i}\right)}^{2}}{N}},\;\;\;\;\;\;where\;y_{i}:real\;values\;and\;{\hat{y}}_{i}:\;predicted\;values$$

### 3.1. Machine Learning with Feature Ranking

**Figure 2:**
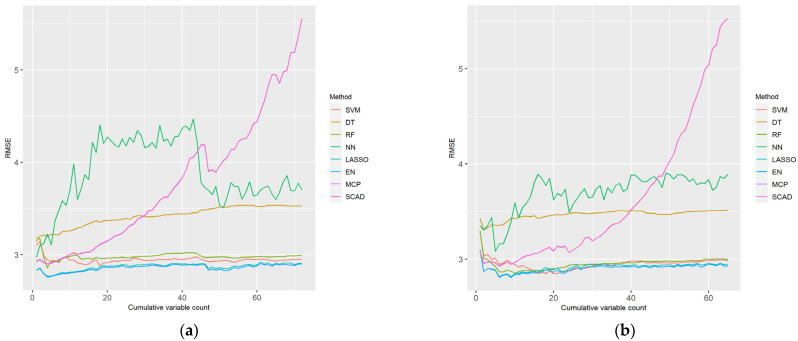
Performance of machine learning (ML) methods with the (**a**) LP and (**b**) XI feature ranking (FR) data for men. The *x*-axis represents the cumulative features according to their rank and the *y*-axis represents the RMSE. The figure legend lists the eight ML methods.

**Figure 3:**
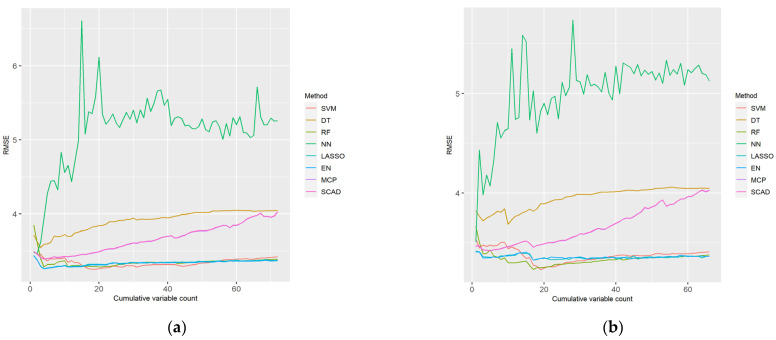
Performance of ML methods with the (**a**) LP and (**b**) XI FR data for women. The *x*-axis represents the cumulative features according to their rank and the *y*-axis reprints the RMSE. The figure legend lists the eight ML methods.

Simple linear regression and XGboost were performed on the cognitive function scores and characteristics, respectively; the data were of men and women aged 75 years or older. Although there were no significant differences in the cognitive function scores, there were differences in the gait and body information data. Therefore, the *p*-value was corrected using FDR. The BH procedure was used as the correction method to avoid extreme data cut-off. The gain represents the relative contribution of a given shape to the model and is calculated based on the contribution of each shape to each tree of the model. When the value of this metric is higher the other features, it is important to generate predictions. Figure 2 and Figure 3 illustrate the RMSE obtained using eight ML methods on ranked datasets. The results were compared by replacing the variables with cumulative features. Regardless of whether the data was that of men or women, NN, SCAD, and DT had a relatively high RMSE, which indicates poor performance. SVM, RF, LASSO, EN, and MCP were slightly different, but their corresponding RMSE values exhibited similar trends. 

Considering the distribution of the cumulative feature RMSE based on the LP and XI ranked data for men, SCAD did not exhibit a constant trend, and its value soared. SVM, RF, LASSO, EN, and MCP exhibited similar RMSE distributions, and EN and LASSO had the lowest RMSE values.

Considering the distribution of the cumulative feature RMSE based on the LP and XI ranked data for women, NN had a distinct pattern and its RMSE was relatively higher than that of other ML models. DT and SCAD had a relatively high RMSE, and SCAD exhibited a pattern wherein the RMSE increased as the features accumulated. SVM, RF, LASSO, EN, and MCP exhibited similar RMSE distributions, and SVM and RF had the lowest RMSE values.

**Figure 4:**
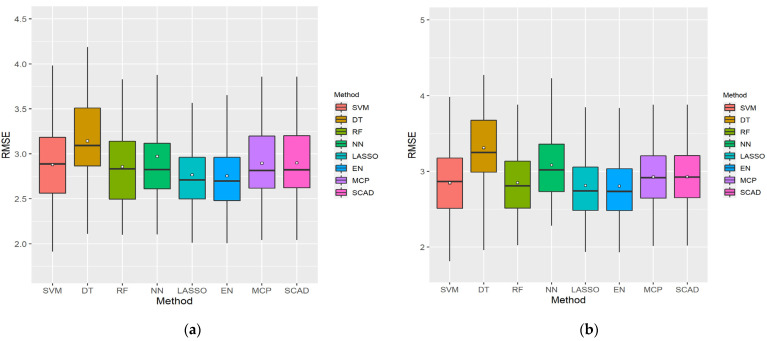
Boxplots of the eight ML methods with cumulative feature count and minimum root mean square error (RMSE) considering the (**a**) LP and (**b**) XI data for men. The bars are the median and the points are the mean. The *x*-axis represents the eight ML methods and the *y*-axis represents the RMSE.

Figure 4 presents boxplots using 100 sampled datasets for cumulative variables with the lowest RMSE in each ML method, considering the LP and XI ranked datasets for men. As shown, DT exhibited a wide RMSE distribution and had the highest error value, whereas EN exhibited a small RMSE distribution and had the lowest error value. SVM had the lowest minimum RMSE values in both cases.

Table 1 lists the RMSE statistics illustrated in the boxplots in Figure 4. As the existing boxplots do not provide the average RMSE values, they were determined separately. The performance of the ML models was compared based on the average RMSE values. The variable size represents the number of cumulative variables. For the LP data, the average RMSE of the five cumulative variables using EN was the lowest, at 2.755567, and its performance was the best. As shown, both LP and XI had the highest error with DT. The SVM of the LP ranking data used the most cumulative variables, with 19, and DT used the least with just two.

**Figure 5:**
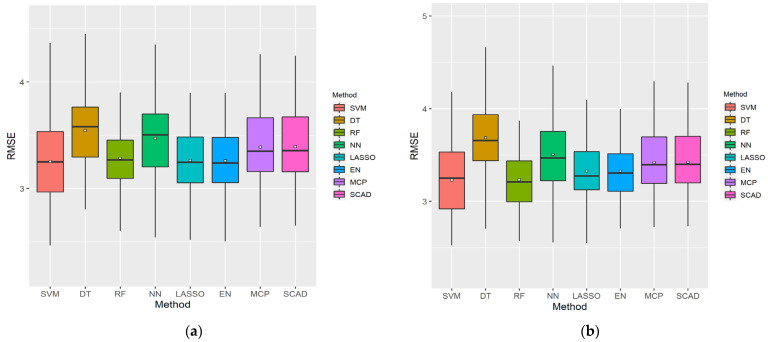
Boxplots of the eight ML methods with cumulative feature count and minimum RMSE considering the (**a**) LP and (**b**) XI data for women. The bars are the median and the points are the mean. The *x*-axis represents the eight ML methods and the *y*-axis represents the RMSE.

Figure 5 presents boxplots using 100 sampled datasets for cumulative variables with the lowest RMSE in each ML method, considering the LP and XI ranked datasets for women. As shown, DT had the highest error value, and EN exhibited the smallest RMSE distribution. SVM had a large RMSE distribution but a minimum RMSE value.

Table 2 lists the RMSE statistics illustrated in the boxplots in Figure 5. As the existing boxplots do not provide the average RMSE values, they were determined separately. The performance of the ML models was compared based on the average RMSE values. The variable size represents the number of cumulative variables. In the XI data, the average RMSE of value of the 20 cumulative variables used with SVM was the lowest, at 3.230709, and the performance was the best. As shown, both LP and XI had the highest error with DT. The RF of the LP ranking data used the most cumulative variables with 21, and the NN of the XI ranking data used the least cumulative variables with 3.

**Figure 6:**
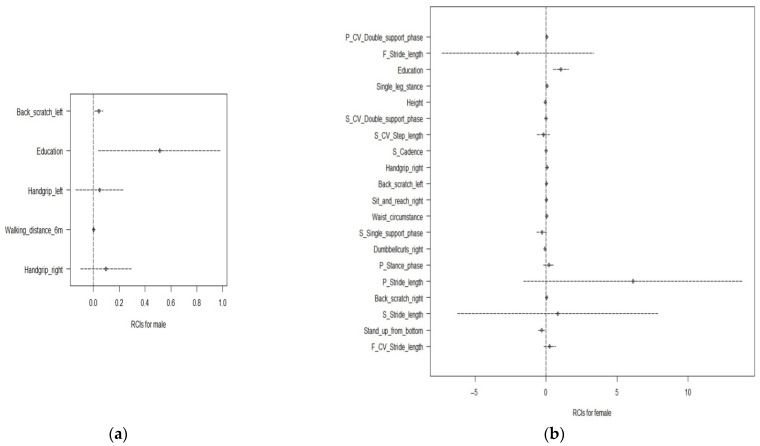
Estimated representation of the coefficients of the optimal model considering the regression coefficient intervals (RCIs) for (**a**) men and (**b**) women. The *x*-axis represents the regression coefficient interval. The *y*-axis represents the significance of five functions selected from the EN of the LP data for men and twenty functions selected from the SVM of the XI data for women.

For men, Education, Handgrip_left, Walking_distance_6 m, Handgrip_right, and Back_scratch_left are the five optimal characteristics that have the highest influence on the cognitive function score, and were obtained from the EN of the LP ranking data. Even with covariance, education is an essential characteristic of the cognitive function scores and has the highest influence, as shown in Figure 6. The beta values of these five features, which exhibited a positive correlation with the cognitive function score, are shown in Table 3.

For women, F_CV_Stride length, Stand_up_from_bottom, S_Stride_length, Back_scratch_right, P_Stride_length, P_Stance_phase, Dumbbellcurls_right, S_Single_support_phase, Waist_circumstance, Sit_and_reach_right, Back_scratch_left, Handgrip_right, S_Cadence, S_CV_Step_length, S_CV_Double_support_phase, Height, Single_leg_stance, Education, F_Stride_length, P_CV_Double_support_phase are the 20 optimal characteristics that have the highest influence on the cognitive function score, and were obtained from the SVM of the XI ranking data. The beta values of these twenty features are shown in Table 4.

### 3.2. Correlation and Network Analyses

**Figure 7:**
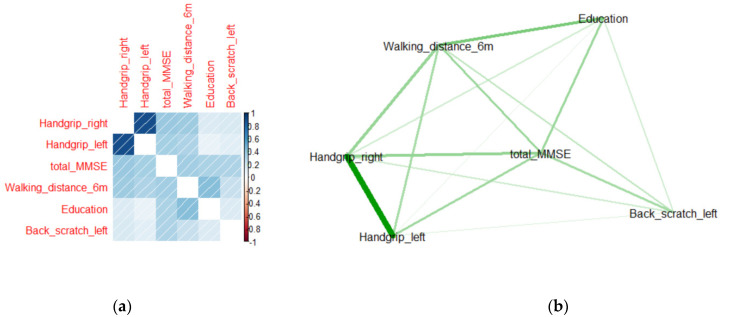
(**a**) Correlogram and (**b**) network analysis of the five optimal features for men. In the correlogram, the blue area represents a positive correlation and the red area represents a negative correlation. The right bar represents the correlation, ranging from −1 to 1. In the network analysis diagram, the green lines represent positive relationships.

**Figure 8:**
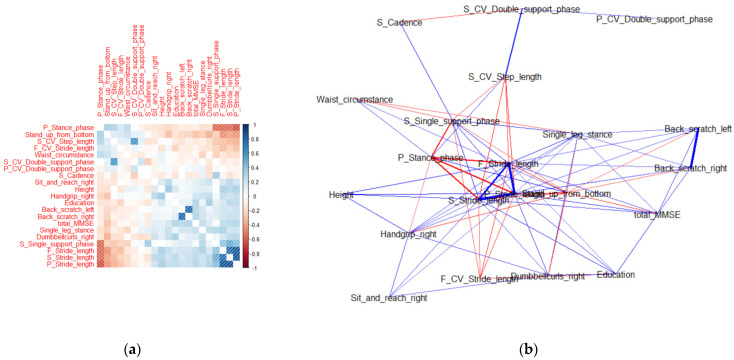
(**a**) Correlogram and (**b**) network analysis of the 20 optimal features for women. In the correlogram, the blue area represents a positive correlation and the red area represents a negative correlation. The right bar represents the correlation ranging from −1 to 1. In the network analysis diagram, the blue lines represent positive relationships and the red lines represent negative relationships.

Correlation analysis is a method of identifying the linear relationship between two variables using a correlation coefficient. Network analysis is a broad and strategic technique used to identify relationships, patterns, and structures between variables [32]. Herein, the optimal features that affect cognitive function scores were determined using eight ML techniques. However, it is difficult to determine whether these features independently affect the cognitive function score or whether they contribute to the cognitive function score through various correlations between them. Therefore, correlation and network analyses were used to identify the correlations between the optimal features and determine the impact of these correlations on the cognitive function score, as shown in Figure 7 and Figure 8.

Considering the optimal characteristics for men, Handgrip_left, and Handgrip_right had the highest correlation in the correlation and network analyses. Education and Walking_distance_6 m had a correlation of approximately 0.45, and Education and Handgrip characteristics had a low correlation. Back_scratch_left had a low correlation with all the other features.

Considering the optimal features for women, P_Stride_length, S_Stride_length, and F_Stride_length had a strong positive correlation with each other, and a strong correlation with other characteristics such as Single_leg_stance, Stand_up_from_bottom, and P_Stance_phase in the network analysis. Back_scratch_left and Back_scratch_right had a weak correlation with the other features but a strong correlation with each other.

## 4. Discussion

### 4.1. Machine Learning

Choosing an appropriate feature to describe a target feature can provide significant performance, time, or cost benefits. Herein, cumulative variables were used by sequentially applying characteristics related to the target characteristics. LP and XI were employed to perform feature ranking to determine the features that influence cognitive function scores based on the body information and gait data of the study participants. LP calculates the individual relationship between each feature and the target feature, whereas XI is an ensemble technique that sequentially modifies predictors and the previous model, and acts as a boosting technique, thereby enabling individual parallel computation of each function [33].

Eight ML models—SVM, DT, RF, NN, LASSO, EN, MCP, and SCAD—were used herein to identify the features with the lowest RMSE for men and women, and to determine the best ML models. As shown in Figure 2 and Figure 3, the higher the number of cumulative features on the *x*-axis, the higher the RMSE. This suggests that feature selection was performed well, and the performance improved when an appropriate number of features was available. For men aged 75 years and older, five features from the LP ranking data were determined as the optimal features that may explain a decline in cognitive function. These optimal features were obtained by performing EN on the LP ranking data. EN combines LASSO with ridges to reduce variance and generate sparse models with a good grouping effect and predictive performance [28]. For women aged 75 years and older, 20 optimal features were determined by an SVM of the XI ranking data. The SVM function selection exhibits good performance when numerous features are available [34]. Therefore, the five optimal features of the LP ranked data and the 20 optimal features of the XI ranked data can adequately predict cognitive function in men and women, respectively, aged 75 years and older.

### 4.2. Important Characteristics of Gait and Physical Fitness to Predict Impaired Cognitive Function

In the model developed herein, the variability domain (CV of all spatiotemporal parameters) of the gait feature is vital to predict declined global cognitive function in older adults. Moreover, the phase domain of the gait feature and cadence were the common variables associated with the optimal features determined herein. Notably, we obtained similar findings in previous studies—an association between increased variability and the phase domain and declined cognitive function [3,4]. The association between increased variability and declined cognitive function may be due to impaired functioning and a reduced hippocampal volume [35]. The increased variability domain may be attributed to an increase in stride-to-stride fluctuations due to the executive dysfunction of cortical sensorimotor control during walking modulation [36]. In addition, the increased phase domain in older adults with declined cognitive function may be attributed to a reduced walking speed in response to inadequate propulsive force during a single support phase [37]. These walking patterns are commonly observed in older adults to compensate for their dynamic instability while walking [3]. Furthermore, a previous study reported that impairments in the cerebellum or basal ganglia may disrupt gait harmony [38], which could also alter gait stability [3]. Gait harmony can be achieved by the golden ratio between the duration of the stance and the swing phase of the gait cycle [39,40]. Therefore, the variability and phase domains are important gait features in predicting a decline in cognitive function in older adults. Our findings on gait features in older women were remarkable since women are more vulnerable than men to declined cognitive function [3]. Indeed, the results of this study using ML strengthen our previous findings.

The important aspects of physical fitness, such as upper- and lower-extremity muscle strength and flexibility, balance, and functional endurance, were highlighted in the results obtained herein. Weakened muscle strength (not only handgrip strength, but also dumbbell curls and ST from an LSP) was associated with declined cognitive function in our previous study [4], as well as other studies [40,41]. This may be related to the decreased gray and white matter volumes in the brain, as well as white matter hyperintensities [42]. Therefore, this result also strengthens our previous findings. However, the influence of important features, such as balance and functional endurance, on declined cognitive function are novel findings and were not determined in our previous studies. Some studies have shown that a poor balance ability could be an indicator of a decline in cognitive function [43,44]. In addition, functional endurance (or exercise capacity) has been associated with memory [45]. Walking requires the efficient integration of multiple neural systems and cognitive subsystems (including orientation, memory, attention, and executive functions) [46]. Balance and endurance are also related to lower gray matter volumes, which is associated with postural instability and poor exercise capacity in older adults, along with lower cognitive function [45,47,48]. Therefore, balance and functional endurance are also important indicators of declining cognitive function.

This study has several potential limitations. First, as the method used herein cumulatively ranks data in order of importance, it does not provide an optimal combination that affects cognitive function scores. Furthermore, variables with an initial increase in error followed by a decrease were assumed to affect cognitive function, whereas variables outside the rankings were ignored. Therefore, further research must be conducted by adding other variables that affect cognitive function. Second, global cognitive function was assessed using only MMSE, which has been shown to have a relatively lower sensitivity [49]. Nevertheless, this screening tool is widely used to evaluate global cognitive function and dementia, including in clinical evaluations.

## 5. Conclusions

LP and XI ranked data were used to select the important features that affect cognitive function scores based on the body information and gait data of 306 study participants. For men, five optimal features were determined from an EN of the LP ranked data. For women, twenty optimal features were determined from an SVM of the XI ranked data. These optimal features of gait and physical fitness should be considered while predicting a potential decline in cognitive function in older adults. The results obtained herein based on a ML approach improve and strengthen previous findings. The optimal features of gait and physical fitness obtained through the proposed ML approach can help improve the prediction of a potential decline in cognitive function. Our results are useful for future works on the dementia prevention. In future studies, additional variables that affect cognitive function can be considered to address the limitations of this study and further improve our understanding of declined cognitive function in older adults.

## Figures and Tables

**Table 1:** Statistical table for cumulative features with minimum RMSE of ranked data for men.

FR	ML	Min	Median	Mean	Max	Variable Size
LP	SVM	1.912	2.887	2.879	3.980	19
	DT	2.109	3.092	3.144	4.187	2
	RF	2.099	2.833	2.857	3.827	5
	NN	2.104	2.823	2.972	8.570	2
	LASSO	2.011	2.709	2.767	3.687	5
	EN	2.006	2.697	2.756	3.654	5
	MCP	2.040	2.814	2.896	3.857	5
	SCAD	2.039	2.822	2.899	3.857	5
XI	SVM	1.815	2.864	2.847	3.982	17
	DT	1.960	3.247	3.310	4.272	3
	RF	2.026	2.809	2.851	3.879	11
	NN	2.281	3.020	3.084	4.891	6
	LASSO	1.936	2.742	2.815	3.847	10
	EN	1.931	2.735	2.806	3.838	10
	MCP	2.015	2.918	2.926	3.880	7
	SCAD	2.019	2.924	2.927	3.880	7

**Table 2:** Statistical table for cumulative features with minimum RMSE of ranked data for women.

FR	ML	Min	Median	Mean	Max	Variable Size
LP	SVM	2.466	3.249	3.253	4.365	19
	DT	2.803	3.579	3.545	4.451	4
	RF	2.473	3.267	3.285	4.173	21
	NN	2.541	3.502	3.470	4.351	3
	LASSO	2.518	3.245	3.263	4.157	5
	EN	2.504	3.239	3.260	4.156	5
	MCP	2.641	3.350	3.389	4.259	5
	SCAD	2.652	3.355	3.393	4.246	5
XI	SVM	2.525	3.250	3.230	4.178	20
	DT	2.705	3.656	3.687	4.664	11
	RF	2.569	3.208	3.233	4.239	18
	NN	2.553	3.467	3.504	5.917	2
	LASSO	2.549	3.273	3.325	4.179	18
	EN	2.706	3.304	3.325	4.173	18
	MCP	2.720	3.394	3.419	4.295	6
	SCAD	2.730	3.399	3.422	4.281	6

**Table 3:** Summary statistics of multiple linear regression for men to see rough each weight of top variables to MMSE scores.

	Beta	Lwr	Upr	SE	t Value	*p*
Handgrip_right	0.097	−0.10	0.29	0.098	0.986	0.326
Walking_distance_6 m	0.003	−0.001	0.01	0.002	1.232	0.221
Handgrip_left	0.048	−0.14	0.23	0.092	0.526	0.599
Education	0.513	0.04	0.99	0.239	2.143	0.034 *
Back_scratch_left	0.041	0.01	0.07	0.016	2.436	0.017 *

Lower bound (lwr); upper bound (upr); standard error (SE); Asterisks denote statistically significant differences at *p* < 0.05.

**Table 4:** Summary statistics of multiple linear regression for women to see rough each weight of top variables to MMSE scores.

	Beta	Lwr	Upr	SE	t Value	*p*
F_CV_Stride_length	0.250	−0.17	0.67	0.214	1.168	0.245
Stand_up_from_bottom	−0.310	−0.54	−0.08	0.114	−2.708	0.007 *
S_Stride_length	0.821	−6.25	7.89	3.583	0.229	0.819
Back_scratch_right	0.032	0.02	0.09	0.027	1.157	0.249
P_Stride_length	6.140	−1.57	13.85	3.907	1.571	0.118
P_Stance_phase	0.198	−0.19	0.58	0.195	1.020	0.309
Dumbbellcurls_right	−0.076	−0.17	0.02	0.048	−1.577	0.117
S_Single_support_phase	−0.300	−0.67	0.07	0.186	−1.612	0.109
Waist_circumstance	0.039	−0.02	0.10	0.030	1.262	0.208
Sit_and_reach_right	0.014	−0.05	0.08	0.030	0.479	0.633
Back_scratch_left	0.021	−0.03	0.07	0.026	0.787	0.432
Handgrip_right	0.065	−0.06	0.19	0.061	1.064	0.289
S_Cadence	−0.008	−0.06	0.04	0.026	−0.333	0.739
S_CV_Step_length	−0.200	−0.65	0.25	0.227	−0.882	0.379
S_CV_Double_support_phase	−0.011	−0.09	0.07	0.041	−0.263	0.793
Height	−0.052	−0.15	0.05	0.050	−1.037	0.301
Single_leg_stance	0.052	0.01	0.09	0.020	2.586	0.011 *
Education	1.040	0.05	1.58	0.275	3.787	0.001 *
F_Stride_length	−2.001	−7.34	3.34	2.705	−0.741	0.459
P_CV_Double_support_phase	0.049	−0.01	0.11	0.032	1.576	0.116

Faster walking speed (F); preferred walking speed (P); slower walking speed (S); coefficient of variance (CV); Asterisks denote statistically significant differences at *p* < 0.05.

## Data Availability

The data presented in this study are available in this manuscript and Table S1.

## Supplementary Materials

The raw data obtained during the study are available online at https://www.mdpi.com/article/10.3390/ijerph182111347/s1. Table S1: Raw data.

## References

[1] Alam U., Riley D.R., Jugdey R.S., Azmi S., Rajbhandari S., D’Août K., Malik R.A. (2017). Diabetic neuropathy and gait: A review. Diabetes Ther..

[2] Tucker M.R., Olivier J., Pagel A., Bleuler H., Bouri M., Lambercy O., del R Millán J., Riener R., Vallery H., Gassert R. (2015). Control strategies for active lower extremity prosthetics and orthotics: A review. J. Neuroeng. Rehabil..

[3] Noh B., Youm C., Lee M., Park H. (2020). Age-specific differences in gait domains and global cognitive function in older women: Gait characteristics based on gait speed modification. PeerJ.

[4] Noh B., Youm C., Lee M., Park H. (2020). Associating gait phase and physical fitness with global cognitive function in the aged. Int. J. Environ. Res. Public Health.

[5] Albers M.W., Gilmore G.C., Kaye J., Murphy C., Wingfield A., Bennett D.A., Boxer A.L., Buchman A.S., Cruickshanks K.J., Devanand D.P. (2015). At the interface of sensory and motor dysfunctions and Alzheimer’s disease. Alzheimers Dement..

[6] Verghese J., Wang C., Lipton R.B., Holtzer R., Xue X. (2007). Quantitative gait dysfunction and risk of cognitive decline and dementia. J. Neurol. Neurosurg. Psychiatry.

[7] Wahid F., Begg R.K., Hass C.J., Halgamuge S., Ackland D.C. (2015). Classification of Parkinson’s disease gait using spatial-temporal gait features. IEEE J. Biomed. Health Inform..

[8] Noh B., Youm C., Goh E., Lee M., Park H., Jeon H., Kim O.Y. (2021). XGBoost based machine learning approach to predict the risk of fall in older adults using gait outcomes. Sci. Rep..

[9] Chtioui Y., Bertrand D., Barba D. (1998). Feature selection by a genetic algorithm. Application to seed discrimination by artificial vision. J. Sci. Food Agric..

[10] Cai J., Luo J., Wang S., Yang S. (2018). Feature selection in machine learning: A new perspective. Neurocomputing.

[11] Lee M., Youm C., Jeon J., Cheon S., Park H. (2018). Validity of shoe-type inertial measurement units for Parkinson’s disease patients during treadmill walking. J. Neuroeng. Rehabil..

[12] Kim Y.K., Joo J.Y., Jeong S.H., Jeon J.H., Jung D.Y. (2016). Effects of walking speed and age on the directional stride regularity and gait variability in treadmill walking. J. Mech. Sci. Technol..

[13] Oyeyemi A.L., Umar M., Oguche F., Aliyu S.U., Oyeyemi A.Y. (2014). Accelerometer-determined physical activity and its comparison with the international physical activity questionnaire in a sample of Nigerian adults. PLoS ONE.

[14] Folstein M.F., Folstein S.E., McHugh P.R. (1975). “Mini-mental state”: A practical method for grading the cognitive state of patients for the clinician. J. Psychiatr. Res..

[15] Chung M.J., Wang M.J. (2010). The change of gait parameters during walking at different percentage of preferred walking speed for healthy adults aged 20–60 years. Gait Posture.

[16] Langhammer B., Stanghelle J.K. (2015). The senior fitness test. J. Physiother..

[17] Jahan S., Khan A. (2012). Power of *t*-test for simple linear regression model with non-normal error distribution: A quantile function distribution approach. J. Sci. Res..

[18] https://3months.tistory.com/262.

[19] Chen T., Guestrin C. XGBoost: A Scalable Tree Boosting System. Proceedings of the 22nd ACM SIGKDD Int Conf on Knowledge Discovery and Data Mining.

[20] Chen C., Zhang Q., Yu B., Yu Z., Skillman-Lawrence P.J., Ma Q., Zhang Y. (2020). Improving protein-protein interactions prediction accuracy using XGBoost feature selection and stacked ensemble classifier. Comput. Biol. Med..

[21] Utkin L.V. (2018). An imprecise extension of SVM-based machine learning models. Neurocomputing.

[22] Somvanshi M., Chavan P., Tambade S., Shinde S.V. A Review of Machine Learning Techniques Using Decision Tree and Support Vector Machine. Proceedings of the 2016 International Conference on Computing Communication Control and Automation (ICCUBEA).

[23] Patil S., Umakant K. Accuracy Prediction for Distributed Decision Tree Using Machine Learning Approach. Proceedings of the 2019 3rd International Conference on Trends in Electronics and Informatics (ICOEI).

[24] Breiman L. (2001). Random forests. Mach. Learn..

[25] Sun Y., Zeng W.D., Zhao Y.Q., Zhang X.M., Shu Y., Zhou Y.G. (2011). Modeling constitutive relationship of Ti40 alloy using artificial neural network. Mater. Des..

[26] Hoerl A.E., Kennard R.W. (1970). Ridge regression: Applications to nonorthogonal problems. Technometrics.

[27] Muthukrishnan R., Rohini R. LASSO: A Feature Selection Technique in Predictive Modeling for Machine Learning. Proceedings of the 2016 IEEE International Conference on Advances in Computer Applications (ICACA).

[28] Zou H., Hastie T. (2005). Regularization and variable selection via the elastic net. J. R. Stat. Soc. Ser. B Stat. Methodol..

[29] Becker N., Toedt G., Lichter P., Benner A. (2011). Elastic SCAD as a novel penalization method for SVM classification tasks in high-dimensional data. BMC Bioinform..

[30] Fan J., Li R. (2001). Variable selection via nonconcave penalized likelihood and its oracle properties. J. Am. Stat. Assoc..

[31] Zhang C.H. (2010). Nearly unbiased variable selection under minimax concave penalty. Ann. Stat..

[32] Emirbayer M., Goodwin J. (1994). Network Analysis, Culture, and the Problem of Agency. Am. J. Sociol..

[33] Ogunleye A., Wang Q.-G. (2019). XGBoost Model for Chronic Kidney Disease Diagnosis. IEEE/ACM Trans. Comput. Biol. Bioinform..

[34] Pal M., Foody G.M. (2010). Feature selection for classification of hyperspectral data by SVM. IEEE Trans. Geosci. Remote Sens..

[35] Annweiler C., Schott A.M., Van Kan G.A., Rolland Y., Blain H., Fantino B., Herrmann F.R., Beauchet O. (2011). The five-times-sit-to-stand test, a marker of global cognitive functioning among community-dwelling older women. J. Nutr. Health Aging.

[36] Beauchet O., Allali G., Annweiler C., Bridenbaugh S., Assal F., Kressig R.W., Herrmann F.R. (2009). Gait variability among healthy adults: Low and high stride-to-stride variability are both a reflection of gait stability. Gerontology.

[37] Taniguchi Y., Watanabe Y., Osuka Y., Kitamura A., Seino S., Kim H., Kawai H., Sakurai R., Inagaki H., Awata S. (2019). Characteristics for gait parameters of community-dwelling elderly Japanese with lower cognitive function. PLoS ONE.

[38] Serrao M., Chini G., Iosa M., Casali C., Morone G., Conte C., Bini F., Marinozzi F., Coppola G., Pierelli F. (2017). Harmony as a convergence attractor that minimizes the energy expenditure and variability in physiological gait and the loss of harmony in cerebellar ataxia. Clin. Biomech..

[39] Iosa M., Fusco A., Marchetti F., Morone G., Caltagirone C., Paolucci S., Peppe A. (2013). The golden ratio of gait harmony: Repetitive proportions of repetitive gait phases. Biomed. Res. Int..

[40] Pentikäinen H., Savonen K., Komulainen P., Kiviniemi V., Paajanen T., Kivipelto M., Soininen H., Rauramaa R. (2017). Muscle strength and cognition in ageing men and women: The DR’s EXTRA study. Eur. Geriatr. Med..

[41] Van Dam R., Van Ancum J.M., Verlaan S., Scheerman K., Meskers C.G., Maier A.B. (2018). Lower cognitive function in older patients with lower muscle strength and muscle mass. Dement. Geriatr. Cogn. Disord..

[42] Callisaya M.L., Beare R., Phan T.G., Blizzard L., Thrift A.G., Chen J., Srikanth V.K. (2013). Brain structural change and gait decline: A longitudinal population-based study. J. Am. Geriatr. Soc..

[43] Bullain S.S., Corrada M.M., Perry S.M., Kawas C.H. (2016). Sound Body Sound Mind? Physical Performance and the Risk of Dementia in the Oldest-Old: The 90+ Study. J. Am. Geriatr. Soc..

[44] Goto S., Sasaki A., Takahashi I., Mitsuhashi Y., Nakaji S., Matsubara A. (2018). Relationship between cognitive function and balance in a community-dwelling population in Japan. Acta Otolaryngol..

[45] Makizako H., Shimada H., Doi T., Park H., Yoshida D., Suzuki T. (2013). Six-minute walking distance correlated with memory and brain volume in older adults with mild cognitive impairment: A voxel-based morphometry study. Dement. Geriatr. Cogn. Dis. Extra.

[46] Scherder E., Eggermont L., Swaab D., van Heuvelen M., Kamsma Y., de Greef M., van Wijck R., Mulder T. (2007). Gait in ageing and associated dementias; its relationship with cognition. Neurosci. Biobehav. Rev..

[47] Kido T., Tabara Y., Igase M., Ochi N., Uetani E., Nagai T., Yamamoto M., Taguchi K., Miki T., Kohara K. (2010). Postural instability is associated with brain atrophy and cognitive impairment in the elderly: The J-SHIPP study. Dement. Geriatr. Cogn. Disord..

[48] Makizako H., Shimada H., Doi T., Park H., Yoshida D., Uemura K., Tsutsumimoto K., Liu-Ambrose T., Suzuki T. (2013). Poor balance and lower gray matter volume predict falls in older adults with mild cognitive impairment. BMC Neurol..

[49] Trzepacz P.T., Hochstetler H., Wang S., Walker B., Saykin A.J. (2015). Relationship between the Montreal Cognitive Assessment and Mini-mental State Examination for assessment of mild cognitive impairment in older adults. BMC Geriatr..

